# Effectiveness and safety assessment of orthopedic device (LSM-01) for low back pain: A randomized, single-blinded, sham-controlled, parallel-group, pilot clinical trial

**DOI:** 10.1097/MD.0000000000028527

**Published:** 2022-01-21

**Authors:** Eun Sol Won, Hyun Lee, Jae Hui Kang

**Affiliations:** a Department of Acupuncture & Moxibustion Medicine, College of Korean Medicine, Daejeon University, Daejeon, Republic of Korea; b Department of Acupuncture & Moxibustion Medicine, Cheonan Korean Medicine Hospital of Daejeon University, Cheonan, Republic of Korea.

**Keywords:** low back pain, LSM-01, muscle relaxation, orthopedic device, pilot trial, randomized controlled trial, study protocol

## Abstract

**Background:**

More than 80% of the population suffer from low back pain at some time during their lives. An orthopedic device (LSM-01) will be used to alleviate back pain caused by muscle tension. LSM-01, which has a rotating roller, stimulates meridian-muscles around the governor vessel, bladder meridian, and gall bladder meridian.

**Methods:**

This study will be a randomized, single-blinded, sham-controlled, parallel-group, pilot clinical trial. Subjects will be randomly allocated to the treatment group (LSM-01) or the control group (sham device). The duration of the clinical trial will be 2 weeks. The primary outcomes will be measured using the visual analog scale; the secondary outcomes will include pressure pain threshold, Oswestry Disability Index, and Patient Global Impression of Change. Statistical analysis will be performed for the full study population. Analysis of covariance will be conducted to identify differences in pain before and after the application of the device.

**Discussion:**

This clinical trial will evaluate the safety and efficacy of the LSM-01 device. As a pilot study, this investigation includes a limited number of subjects. The results of this pilot trial will form a basis for a large-scale clinical trial, which will be conducted in the future.

**Clinical trial registration:**

This study protocol is registered with the Clinical Research Information Service (CRIS) of Korea. Clinical trial registration number: CRIS-KCT0006425. Registered: October 5, 2021; https://cris.nih.go.kr/cris/search/detailSearch.do?search_lang=E&search_page=L&pageSize=10&page=undefined&seq=20056&status=5&seq_group=20056

## 1 Introduction

Back pain is a musculoskeletal abnormality (excluding pain caused by pregnancy or infection), which persists for >3 days in the waist area of the thoracic spine.^[1]^ Chronic back pain refers to pain which persists for >3 months. Pain continues to occur even after the primary damaged tissue has healed.^[2]^ Spinal pain is attributed to numerous reasons, such as severe exercise or accident, spinal disease, aging, wrong posture, and lack of exercise.^[3]^ When resistance to gravity is compromised, the human body attempts to compensate; twisting of the spine changes the gravitational line, leading to an unstable posture. Subsequently, exposure of the musculoskeletal system to more load on 1 side can lead to pain.^[4]^ Most individuals experience spinal pain at least once in their lives. A study reported that the annual prevalence of spinal pain ranges 15% to 45% and 30% on average.^[5]^

In Korean Medicine, governor vessel (GV), bladder meridian (BL), and gall bladder meridian (GB) are important treatment target for chronic low back pain (LBP).^[6]^ The efficacy of various Korean Medical interventions, such as Thread Embedding Therapy^[7]^ and Warm Needle Acupuncture,^[8]^ for LBP are studied using these meridians. These meridian-muscles correspond to the muscular system, such as spinous process, erector spinae muscle, gluteus medius, and tensor fasciae latae. In addition, these muscles are used in the myofascial release technique of Chuna manual therapy to treat chronic LBP.^[9,10]^

LSM-01 has a rotating roller that uses electricity to stimulate the meridian-muscles around GV3, GV4, GV6, GV9, GV12, GV14, GB30, GB31, GB32, GB33, BL13, BL17, BL20, BL23, BL25, BL26, BL42, BL46, BL49, and BL52. It relieves muscle stiffness through mechanical stimulation. The objective of this pilot clinical trial is to evaluate the safety and efficacy of LSM-01 in subjects with LBP compared to sham device.

## 2 Methods

### 2.1 Study design and sample size calculation

**Figure 1:**
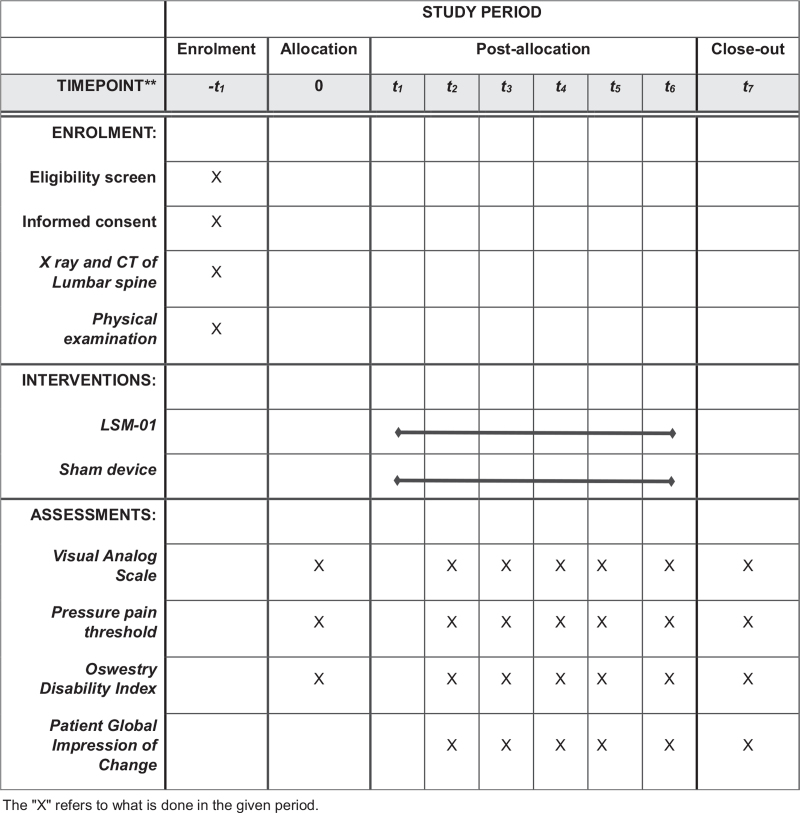
Standard Protocol Items: Recommendations for Intervention Trials (SPIRIT) figure show schedule of enrolment, interventions, and assessments. The “X” refers to what is done in the given period.

This study was designed as a randomized single-blinded, sham-controlled, parallel-group, pilot clinical trial. The protocol ver 1.1 is approved by the Institutional Review Board (IRB) on July 1, 2021. A total of 30 subjects with LBP will be recruited from the DUCKMH through Banner promotion; the recruitment commenced in June 2021. All subjects will receive a written explanation of the study protocol and an informed consent form by investigator. X-ray and computed tomography (CT) imaging of the L-spine will be performed for those with LBP based on history or physical examination. Additionally, vital signs will also be measured at every visit. For preliminary and parallel-design studies, Julious suggested that the empirically optimal sample size is 12 individuals per group, considering feasibility, estimated average, and dispersion accuracy.^[11]^ Considering a dropout rate of 20%, the calculated sample size for this pilot clinical trial is 30. A total of 30 patients with LBP who meet the eligibility criteria will be randomly assigned to groups in a 1:1 ratio. According to their group, the subjects will be treated with the LSM-01 device or sham device for 2 weeks. The total duration of the clinical trial is 3 to 4 weeks. The total number of visits is 7, and the efficacy and safety of the treatment are assessed at each visit. Subjects are notified of their further visit schedule by the investigator each visit. The administration of pain relievers will be maintained at the same dose and frequency until the end of the clinical trial. The study flow chart is presented in Table 1 and Figure 1.

**Table 1:** Schedule for the enrollment, intervention, and assessments.

	Study period				
	Recruitment	Intervention period			Observation period
Visit	Screening	V1 Before intervention	V1 After intervention	V2–V6	V7
Checking the selection/exclusion criteria	•				
Vital signs	•	•		•	•
Demographic surveys and body measurements	•				
Medical history of lumbar and other body organs	•				
Lumbar spine X-ray and computed tomography (CT)	•				
Physical examination	•				
Random assignment		•			
Treatment (Orthopedic or sham device)		•		•	
Visual Analog Scale	•	•		•	•
Pressure pain threshold		•		•	•
Oswestry Disability Index		•		•	•
Patient Global Impression of Change				•	•
Checking medical history and changes in treatment drugs			•	•	•
Suspending the test and checking the criteria for elimination			•	•	•
Safety assessment			•	•	•

### 2.2 Inclusion and exclusion criteria

The inclusion criteria are as follows: 20 to 70 years of age; visual analog scale (VAS) ≥75 mm; and use of painkillers. The exclusion criteria are as follows: history of spinal fractures; spinal surgery; severe back pain with VAS >75 mm lasting >3 days within 3 months; neurological symptoms of sensory and motor paralysis; abnormalities detected on L-spine X-ray and CT examination; severe pain in other parts of the body; skin diseases; inflammation; presence of wounds around the hip joint of the spine and pelvis; other treatment for back pain except painkillers; cardiovascular disease; tumors; alcoholism; drug abuse; and participation in other clinical trials within 30 days.

### 2.3 Randomization and blinding procedures

An independent statistician will randomly assign 15 individuals per group using the statistical program SAS Version 9.4 (SAS Installation. Inc., Cary, NC). Subjects will be assigned using balanced block randomization, without stratification. The clinical trial manager will assign screening numbers to subjects in the order they have provided informed consent. Subjects will be assigned to the experimental group or control group according to their randomization identification code. The information regarding the assignment of intervention will be stored in an independent secure place. The randomization code will be placed in an opaque envelope. The trial investigator will not perform the randomization and assignment of intervention. Therefore, subjects and investigators are blinded. Moreover, the trial investigator will not serve as the operator in this study.

### 2.4 Device

**Figure 2:**
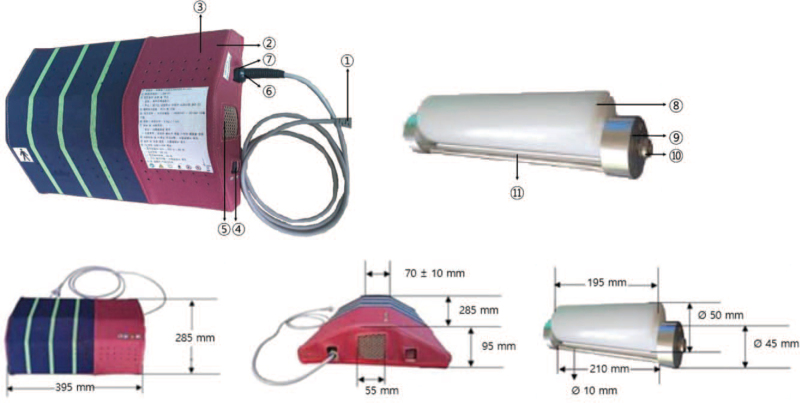
Appearance and size of the device.

**Figure 3:**
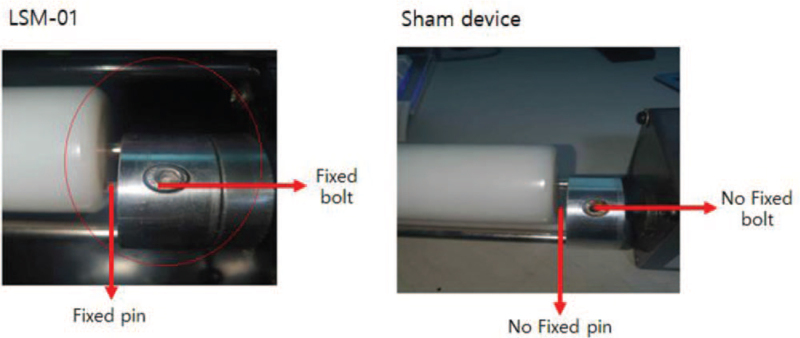
Differences between the LSM-01 and sham device: no fixed bolt and pin.

The device used in this clinical trial has an electric motor (Fig. 2, Table 2). This second-class medical device is licensed by the Ministry of Food and Drug Safety of Republic of Korea for the purpose of relieving lumbar muscle pain (classification number: A67025.01). We conduct clinical trials using this device to evaluate its safety and efficacy. There is no difference between the LSM-01 and sham device in terms of appearance, vibration, and sound. However, there is a difference in the rotation of the roller (Fig. 3). In the LSM-01, the connection axis between the motor unit and the rotating roller is connected normally. In the sham device, rotational fixing pins and fixed hexagonal pins are missing and prohibit rotation. The feel of the LSM-01 and sham device is similar and blinding is maintained. The LSM-01 and sham device are manufactured by Spine Muscle Strengthening Machine Co. (Namyangju, Republic of Korea).

**Table 2:** Appearance and description of the device.

(1)	Power cord	Connects power to the main board
(2)	Body cover	For safety, a cover protecting the user's contact part
(3)	Exhaust vent	Ventilates the interior of the device
(4)	Power switch	Turns on/off the device
(5)	Body cover	For safety, a cover protecting the user's contact part
(6)	Cable holder	Fixes the power cable
(7)	Current fuse	Protects the product from excessive current
(8)	Roller	Acts mechanically by direct contact with the human body
(9)	Rotating core	Rotates the roller continuously
(10)	Bearing	Rotates the roller more softly
(11)	Support	Separates roller contact with the human body in each rotation

### 2.5 Intervention

Firstly, while the patient is lying in the lateral position, the LSM-01 will be applied to the gluteus medius 30 times (60 s/application). Secondly, in the same position, the LSM-01 with be applied to the tensor fasciae latae 15 times (30 s/application). Finally, while the patient is lying in the supine position, the LSM-01 will be applied to the spinous process of S1, L5, L3, L1, T11, T8, T4, and C7 15 times (30 s/application). The intervention will be repeated twice. A single qualified Korean medical doctor will perform the procedure using the device in both groups. All eligible subjects will receive treatment according to their group (i.e., LSM-01 or sham device). The treatment will be performed thrice weekly for a total of 6 times. During the trial, the use of painkillers will be allowed, whereas other treatments are prohibited.

### 2.6 Outcome measures

The primary outcome is the VAS score, which will be used to assess LBP at each visit. The secondary outcomes are the average change in the pressure pain threshold (PPT), Oswestry Disability Index (ODI) score after treatment (V7) compared with baseline (V1), and Patient Global Impression of Change (PGIC). VAS, PPT, ODI, and PGIC will be measured at each visit.

#### 2.6.1 VAS

The VAS is used to evaluate the intensity and frequency of pain. The visual reflection scale is used by the patient himself, indicating the degree of pain on a 10-cm horizontal straight.

#### 2.6.2 PPT

The PPT is determined by pressing vertically on both sides (BL25) of the subject at a rate of 1 kg/s with a pressure algorithm and measuring the pressure (kg/cm) at the moment the subject first complains of pain.

#### 2.6.3 ODI

The ODI indicates dysfunction due to pain. It consists of 6 questions for 10 items, total of 60 question. For each question, subjects use a score from 0 to 5 points, with higher scores indicating greater disability.

#### 2.6.4 PGIC

The PGIC evaluates the improvement perceived by the subject after treatment versus baseline using the following answers: “Substantially better,” “A lot better,” “A little better,” “No change,” “A little worse,” “A lot worse,” and “Very much worse.”

### 2.7 Data collection and monitoring

At screening, the subjects will complete a questionnaire regarding their sociodemographic characteristics, provide their medical history, and undergo X-ray and CT examinations of the lumbar. When the dropout occurs, outcomes are obtained as possible. Personal information and data will be managed by the investigators. The final trial dataset will be accessible to statisticians and principal investigator (PI) and can be accessible with the permission of the PI. All information regarding the subjects and intervention will be kept confidential. All documents related to the clinical trial will be recorded and classified using an identification code, rather than the name of the subject. An independent contract research organization (CRO) – MEDI CRO Co., Ltd. (Seoul, Republic of Korea) – will monitor the research plans, case records, and annexed documents according to the schedule included in the clinical trial protocol. CRO has no competing interest. The CRO will review all evidence documents related to the clinical trial and perform data quality control. At the end of the trial, the case report forms will be stored in a locked cabinet in accordance with IRB regulations.

### 2.8 Statistical analysis

Data analysis, including the validity evaluation variable, will be performed in the full analysis set group. The per protocol group analysis will be performed as the supplementary analysis. An analysis of covariance using the baseline value of VAS as a covariate will be performed. The ambient mean and standard error, 95% confidence interval, *t* statistic, and *P*-value for change in each group will be determined. Variables for the evaluation of efficacy will also be analyzed in the same manner. In all statistical tests, an α = 0.05 on both sides will indicate a statistically significant difference. All statistical analyses will be performed using the SPSS Statistics for Windows Version 20.0 (IBM Corp., Armonk, NY) software. Last observation carried forward analysis will be used to handle missing data. Safety evaluation will be mainly performed by analyzing the frequency of adverse events (AEs) and serious adverse events (SAEs), which are suspected to be associated with the treatment. The evaluation of safety will be conducted in the safety population. An independent sample *t*-test or Wilcoxon rank-sum test will be performed for the analysis of continuous variables. The McNemar test will be used for the analysis of categorical variables. A chi-squared test or Fisher exact test will be performed to examine the potential association of the treatment with the occurrence of AEs.

### 2.9 Safety

AE refers to all harmful and unintended reactions caused by medical devices used in clinical trials. Local reactions (predictable AEs) that may occur after treatment with medical devices in clinical trials include bruises, local pain, and burning. An AE that meets the criteria for a SAE will be reported to the IRB. In the case of SAE, appropriate solutions for the number of uses or intensity will be taken by the PI's judgment immediately. If necessary, unblind could be considered by IRB. The occurrence of all AEs will be assessed at each visit. The number and percentage of subjects who experience at least 1 AE will be calculated. Symptoms, date of onset, and duration of expected AEs will be recorded.

### 2.10 Ethics

This study was designed referencing the Helsinki Declaration and the Korean Clinical Practice Guidelines, and has been approved by the IRB of DUCKMH (number DJUMC-2021-MD-01-1). This study protocol is registered with the Korean National CRIS (CRIS-KCT0006425). In case modifications to the protocol are to be carried out, these modifications will be approved by the IRB. Subjects may be required to withdraw from the study in case of SAEs, and these SAEs will be reported to the IRB. Prior to enrollment, interviews with the subjects will be conducted by a Korean medical doctor to evaluate their suitability for participation in this clinical trial. Monitor will check the records of subjects for the verification of the reliability of clinical trial procedures and data. All data processing will be performed in accordance with the relevant regulations and subject confidentiality will be assured.

Following the occurrence of a SAE, the trial investigator will contact the PI to obtain consent for the release of the blinding. Moreover, this process will be recorded in writing and reported to the IRB within 24 hours. Harmful and unintended reactions during clinical trials of medical devices are compensated in accordance with the Victim Compensation Protocol.

## 3 Discussion

More than 80% of the population will suffer from LBP at some point in their lives.^[12]^ For most, the clinical course is benign, with 95% of those afflicted recovering within a few months of onset.^[13]^ However, some will develop chronic LBP. Recurrences of LBP are also common; the incidence rates of subsequent LBP episodes within 1 year for working populations and throughout the lifetime of an individual are 20% to 44% and ≤85%, respectively.^[14]^ For these reasons, there is a growing interest in the development of effective and safe medical devices for the treatment of LBP.

Thus far, this is the first study investigating the effectiveness and safety of LSM-01 (orthopedic machinery) in the treatment of LBP. Because this is a pilot study, the sample size is small and insufficient to yield concrete conclusions regarding the effectiveness of LSM-01. Nevertheless, this pilot clinical trial may provide insights into this treatment modality and evidence for future large-scale clinical trials that will investigate the therapeutic effects of the LSM-01 in individuals with LBP.

## Author contributions

**Conceptualization:** Jae Hui Kang.

**Resources:** Eun Sol Won, Jae Hui Kang.

**Supervision:** Jae Hui Kang.

**Visualization:** Hyun Lee.

**Writing – original draft:** Eun Sol Won.

**Writing – review & editing:** Hyun Lee, Jae Hui Kang.
